# Urinary Proteins of Female Domestic Dog (*Canis familiaris*) during Ovarian Cycle

**DOI:** 10.3390/vetsci10040292

**Published:** 2023-04-14

**Authors:** Martyna Woszczyło, Paweł Pasikowski, Sankarganesh Devaraj, Agata Kokocińska, Antoni Szumny, Marcin J. Skwark, Wojciech Niżański, Michał Dzięcioł

**Affiliations:** 1 Department of Reproduction and Clinic of Farm Animals, Wroclaw University of Environmental and Life Sciences, Plac Grunwaldzki 49, 50-366 Wrocław, Poland; 2 Captor Therapeutics Inc., Duńska 11, 54-427 Wrocław, Poland; 3 Department of Biotechnology, School of BioSciences and Technology, Vellore Institute of Technology, Vellore 632014, India; 4 Institute of Biological Bases of Animal Production, University of Life Sciences in Lublin, 13 Akademicka St., 20-950 Lublin, Poland; 5 Department of Chemistry, Wroclaw University of Environmental and Life Sciences, C.K. Norwida 25, 50-375 Wrocław, Poland; 6 InstaDeep Ltd., 5 Merchant Square, London W2 1AY, UK

**Keywords:** dogs, urine, proteome, estrus, chemical signals, LC–MS/MS

## Abstract

**Simple Summary:**

Chemical signals are essential for communication between living organisms. Dogs possess two sensory organs enabling chemical communication: the main olfactory system and the vomeronasal organ (VNO). Additionally, contact chemoreception is also pertinent, by which non-volatile molecules, including but not limited to proteins, are recognized as chemical signals. However, non-volatile chemical signals have been sparsely studied in dogs. Therefore, we aimed to examine the urinary proteins of female domestic dogs during the estrus and anestrus phases to detect and identify such non-volatile chemical signals.

**Abstract:**

The presence and identity of non-volatile chemical signals remain elusive in canines. In this study, we aim to evaluate the urinary proteins of female domestic dogs in the estrus and anestrus phases to evidence the presence of non-volatile chemical signals and to elucidate their identities. We collected urine samples from eight female dogs in the estrus and anestrus phases. A total of 240 proteins were identified in the urine samples using liquid chromatography–mass spectrometry (LC–MS analysis). The comparison of the proteins revealed a significant difference between the estrus and anestrus urine. We identified proteins belonging to the lipocalin family of canines (beta-lactoglobulin-1 and beta-lactoglobulin-2, P33685 and P33686, respectively), one of whose function was the transport of pheromones and which was present only in the estrus urine samples. Moreover, proteins such as Clusterin (CLU), Liver-expressed antimicrobial peptide 2 (LEAP2), and Proenkephalin (PENK) were more abundant in the estrus urine when compared to the anestrus urine. LEAP2 was recently described as a ghrelin receptor antagonist and implicated in regulating food intake and body weight in humans and mice. Proenkephalin, a polypeptide hormone cleaved into opioid peptides, was also recognized as a candidate to determine kidney function. As of yet, none of these have played a role in chemical communication. Clusterin, an extracellular chaperone protecting from protein aggregation implicated in stress-induced cell apoptosis, is a plausible candidate in chemical communication, which is a claim that needs to be ascertained further. Data are available via ProteomeXchange with the identifier PXD040418.

## 1. Introduction

Communication using chemical signals is widespread in the world of living organisms. A variety of information regarding different physiological and pathological conditions in animals is transmitted through this mode [1,2]. Semiochemical communication using sex pheromones is one of the best-known examples of this mechanism. In dogs, sex pheromones secreted by a female during the proestrus and estrus phases are responsible for the modulation of physiology and behavior of male dogs [3,4,5,6,7]. While the composition of volatile compounds in the urine during various stages of the ovarian cycle in female canines has been extensively studied, current studies also emphasize the role of less volatile compounds [7,8,9,10,11]. Such compounds, present in the female secretions and collected by males during direct contact with the female (licking a vulva), may be involved in inter-individual communication and recognition of sexual attractiveness [11].

Like many other vertebrates, dogs use two sense organs for chemical communication: the main olfactory system and the vomeronasal organ (VNO). Both of these organs were reported to function in chemical signaling; however, the molecules perceived by the two systems vary. For instance, the MOS detects general odorants; whereas, the VNO, in some species, is reported in the detection of pheromones. However, in dogs, the vomeronasal type 2 receptor is missing, which raises the question of whether the VNO is functional [1,2,12,13,14]. Evaluation of the detected chemical compounds (including odors) helps assess the reproductive status of the signal emitter. It is also important to note that the chemical signals are used by the estrus females to attract males. However, exhibition of consummatory sexual behaviors is a multi-step process [6]. Similar to other species, male dogs approach, sniff, and lick the vulva of the females. The peculiar behavior is that the males also lick the urine of females [7,11,15,16], which could facilitate the transfer of volatile and non-volatile compounds. Indeed, the volatile and non-volatile compounds play an important role in estrus detection [11].

Urinary proteins are unequivocally vital in chemical communication. For instance, MUPs and α2u-globulins are the best-studied chemical signals reported in the urine of mice and rats, respectively. MUPs are lipocalins (proteins that transport small hydrophobic molecules) that bind with pheromones [13]. In mice, urine-derived scent marks have multiple roles in communication, mainly because of MUPs and other pheromones [17]. MUPs provide stability to the bound pheromones and can also function as pheromones themselves. It has been shown that MUPs modulate the behavior of males in that it promotes inter-male aggression [18,19]. Additionally, MUPs also stimulate reproductive behaviors and accelerate puberty attainment in females. Some of these functions are postulated as not being unique to rats and mice alone [20]. Mentioned above, Logan et al. [20], in evaluating parallel expansions of non-rodent MUP clusters, found that apart from other species, such as pigs, baboons, chimpanzees, bush babies, and orangutans, dogs also have a single MUP gene. Studies on rats by Rajkumar et al. [21] detected alpha (2u)-globulin and demonstrated its functional analogy in the Indian commensal rat (*Rattus rattus*). Changes in the composition and concentration of the volatile compounds are considered to develop estrus detection methods in some species [10,22,23,24,25]. Evaluation of the urinary protein profile (unique proteins at a particular phase or the difference in the concentration) was also conducted in several species [25,26,27,28,29]. However, information about urinary proteomes that may have implications in estrus detection is scanty for domestic dogs.

Ferlizza et al. [30] stated that urine is an ideal sample for the studies of proteomics and metabolomics; however, specific urinary biomarkers are currently lacking in dogs. The urinary proteins were investigated in regard to kidney diseases, emphasizing the potential of a dog as an animal model for human disease states [28,30,31]. A recent study [32] also stated that the body fluids of dogs still need to be better characterized in the context of proteomic studies.

The serum proteome was mapped using 2D PAGE in humans [33], horses [34], and dogs [35]. Altherton et al. [36] also characterized the canine serum proteome using mass spectrometry; wherein, 32 proteins were identified, and the reference ranges for albumin and globulin sub-fractions were established in 17 dogs. In a subsequent study, the changes in the canine serum proteome were attributed to the homeostatic disturbance resulting from various diseases [37]. Interestingly, Szczubiał et al. [38] described plasma proteins in pregnant and non-pregnant female dogs. In contrast, Dąbrowski and Franco Martínez described the roles of blood and salivary acute proteins in the condition known as pyometra [39,40]. Despite this, neither urinary proteomes nor reproduction-related aspects of proteomes have been investigated in canines.

Apart from blood, other body fluids (e.g., cerebrospinal fluid (CSF), follicular fluid), as well as tissues (e.g., parietal cortex) were investigated in dogs in the context of proteome evaluation [41]. In the aforementioned study, in both genders, the urine contained proteins derived from the serum through ultrafiltration and incomplete reabsorption. For instance, albumin was a predominant protein in the urine. Authors also identified unique proteins in the urine of males and females, confirming the sexual dimorphism urinary proteins in canines. Teinfalt et al. [42] identified a prostate-specific protein (PSP; 30 kDa) in the urine of intact males, which was absent in females and castrated males. This study indicated that the composition of the urine collected during natural micturition and cystocentesis varied and underscored the involvement of the prostate gland in regulating the urinary constituents.

Studies on chemical communication in canines hitherto mainly focused on volatile compounds. Taking this into account, in this study, we aimed to evaluate the changes in the composition of the female canine urinary proteome during various stages of the ovarian cycle to ascertain any relevant biomarkers of estrus.

## 2. Materials and Methods

### 2.1. Ethics Statement and Animals

The study was conducted following the regulations on animal experimentation and guidelines for the use of animals in research. The experimental protocol was approved by the 2nd Local Commission for Animal Experimentation in Wrocław, Poland (permission no. 17/2017). The animals used in the experiments were Beagle breed dogs belonging to the Local Experimental Kennel and patients of the local clinic of reproduction.

### 2.2. Sample Collection

#### Urine Sampling

Midstream urine samples from the healthy female donor dogs were collected in the morning during spontaneous urination using a sterile steel ladle. Samples were stored in glass vials at −20 °C. The experiment was conducted with eight individuals, and twelve samples were collected. The samples after pooling constituted nine experiments:

- One animal in estrus only; single sample (Female 1).
- Two animals in estrus; two samples each (Female 2 and Female 3):
  ○ One estrus sample of Female 3 contributed to pool P1.
  ○ Additional sample of Female 2 in anestrus, out of which a portion was analyzed independently and the remaining contributed to pool P2.
- One animal in estrus and anestrus; one sample each (Female 4):
  ○ Estrus sample was used as part of pool P1.
  ○ Due to low volume, anestrus sample was used as part of pool P2.
- Four immature ones (constituting experimental pools P3: Female 5 and Female 6; P4: Female 7 and Female 8).
- Exact sample coding is described in Supplementary Materials along with the specific protein results. Sample scheme is also further described in Table 1.

### 2.3. Determination of the Phases of Cycle

The estrus cycle stages’ determination was based on vaginal cytology and plasma progesterone concentrations. Additionally, the consummatory sexual behaviors of male dogs toward females and owners’ observations of the female dogs were also considered [10,43].

#### 2.3.1. Vaginal Cytological Examination

Vaginal smear samples were stained with Haemacolor^®^ (Merck KGaA, Darmstadt, Germany) stain, and based on the percentage of the cornified superficial cells, the estrus cycle stages were evaluated [44].

#### 2.3.2. Progesterone Level Evaluation

The plasma progesterone concentration was determined by an enzyme-linked fluorescence assay (ELFA; mini VIDAS^®^ Biomerieux, Marcy-L’étoile, France) with the mean level of progesterone 21.66 ng/mL (with a standard deviation of 0.823 ng/mL, i.e., coefficient of variation (CV) of 3.8%) [45].

### 2.4. Proteomic Approach

#### 2.4.1. BCA Assay

The urine samples (*n* = 18) were centrifuged (1000× *g*, 10 min, 4 °C) to remove any insoluble particles and cell debris. The resulted supernatants were diluted 10-fold with 2 M urea and stored as aliquots of 1.5 mL at −80 °C to use in protein measurement. Protein concentration in the urine samples was determined using Pierce™ BCA Protein Assay Kit (Thermo Scientific, Waltham, MA, USA).

#### 2.4.2. Sample Pooling

Some samples from the same stage (either estrus or anestrus) were pooled to facilitate experiments; whereas, several samples were analyzed individually. This also created the reference sample, which was used in every run for inter-run comparison and scaling.

#### 2.4.3. Reduction, Alkylation, Digestion, and Tandem Mass Tag (TMT) Labeling

FASP (Filter-Aided Sample Preparation) was used to prepare samples for mass spectrometric analysis. In FASP, a mass weight cut-off of 3 kDa was used. The urine samples containing 80 µg of protein were treated with 8 M urea (Sigma-Aldrich, St. Louis, MO, USA) and 10 mM DTT (dithiothreitol, Sigma-Aldrich) (prepared in 100 mM trimethylammonium bicarbonate (TEAB)) for 1 h at 55 °C. Subsequently, the samples were alkylated with 17 mM iodoacetamide (Thermo Scientific) in 100 mM TEAB at room temperature for 30 min. After incubation, the samples were transferred to cut-off membrane-fitted tubes and centrifuged at 10,000× *g* for 30 min. The resulting flow-through was collected from the lower portion of the tube and digested with trypsin (sample: trypsin at a ratio of 40:1) in 50 mM TEAB (Promega) at 37 °C for 18 h. The peptides were labeled with the TMTsixplex (Tandem Mass Tags) Label Reagent Set (Thermo Scientific) according to the manufacturer’s instructions. The resulting samples were stored at −80 °C until further processing. All the samples were prepared in triplicates. To facilitate comparison between samples, we included the samples from Pool 1 in both TMT six-plexes. The sample division across six-plexes can be found in Supplementary Table S2.

#### 2.4.4. LC–MS/MS

All the measurements were performed using LTQ Elite Orbitrap ETD (Thermo Scientific, USA) connected to the Easy nLC 1000 chromatograph (Thermo Scientific) at the Mass Spectrometry Laboratory of Łukasiewicz Research Network, Polish Center for Technology Development, Wrocław, Poland. Peptides were trapped using Acclaim PepMap C-18, 2 cm trap column (Thermo Scientific) and separated on Acclaim PepMap C-18 column (100 Å, 500 mm × 0.075 mm × 3 μm) (Thermo Scientific) in ambient temperature. Mobile phases A and B were 0.1% formic acid in water and in acetonitrile: water [90:10 (*v*/*v*)], respectively. Five microliters of the sample was injected at a flow rate of 300 nL/min with a gradient from 2 to 55% of phase B (during 200 min). The external calibration of mass spectrometer with LTQ Velos Positive calibration standard was employed, achieving the standard deviation of measurements below 1 ppm. Measurements were performed in positive ion mode with *m*/*z* ratio between 110 and 2000. The capillary voltage was 3 kV. Higher energy collisional dissociation (HCD) fragmentation of top 10 peaks was employed with normalized collision energy set to 35 eV in 1 *m*/*z* isolation window with minimum 2+ charge state of parent ion and dynamic exclusion for 30 s after two spectra.

#### 2.4.5. Protein Identification and Quantification

Mass spectra were processed with Proteome Discoverer 2.4, and Sequest HT search engine was used to extract and annotate MS/MS spectra. To assign spectra to canine proteomes, we conducted a Sequest HT search against a custom database, which comprised SwissProt and TrEMBL protein sequences of *Canis lupus familiars* and its subspecies, which were extracted from UniProtKB 2022_12. Trypsin was selected as an enzyme with a maximum of two missed cleavages. Maximum precursor and fragment tolerances were set to 20 ppm and 0.1 Da, respectively, to permit for higher sensitivity of the experiment. We also explored a more stringent set of thresholds of 10 ppm/0.05 Da, as well as 10 ppm/0.02 Da. Modifications were set as follows: quantification—TMT 6plex; static modification—carbamidomethyl (C); and dynamic modifications—acetyl (protein N-term) and oxidation (M). Reporter ion quantification was performed using Proteome Discoverer 2.4. Spectra were normalized to the total amount of peptides in the sample and scaled to the reference sample channel (TMT 126 in urine samples). Unique and razor peptides were used for quantification with at least two peptide matches per protein. The false discovery rate was set to 0.01 (strict) and 0.05 (relaxed).

### 2.5. Statistical Methods

Statistical analysis of collected data was performed using the functionality implemented in Proteome Discoverer 2.5 [46]. Protein FDR rate was assessed at two levels: 0.01 (as a strict criterion) and 0.05 (relaxed criterion). The statistical significance of quantification values for proteins was tested with background-based multiple *t*-testing. This method considers the prior distribution of protein and peptide abundance rates, allowing for the estimation of a relative change for this background. As the samples came from comparable animals, the fundamental assumption of this approach, that the expected protein abundances between samples remain invariant, holds in this case. Protein ratio calculation was performed on protein abundance basis with a maximum fold change of 1000 allowed to avoid unduly confounding effects of outliers. For missing values, low abundance resampling was introduced.

**Figure 1:**
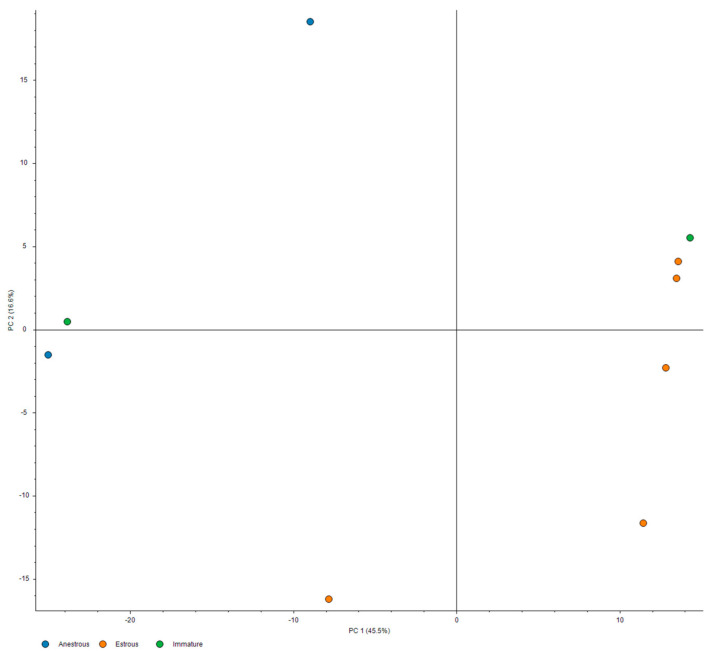
PCA analysis of the identified urinary proteins. The orange circle indicates the proteins from estrus stage, and blue circle indicates the proteins from anestrus urine samples, while immature samples are labeled in green.

**Figure 2:**
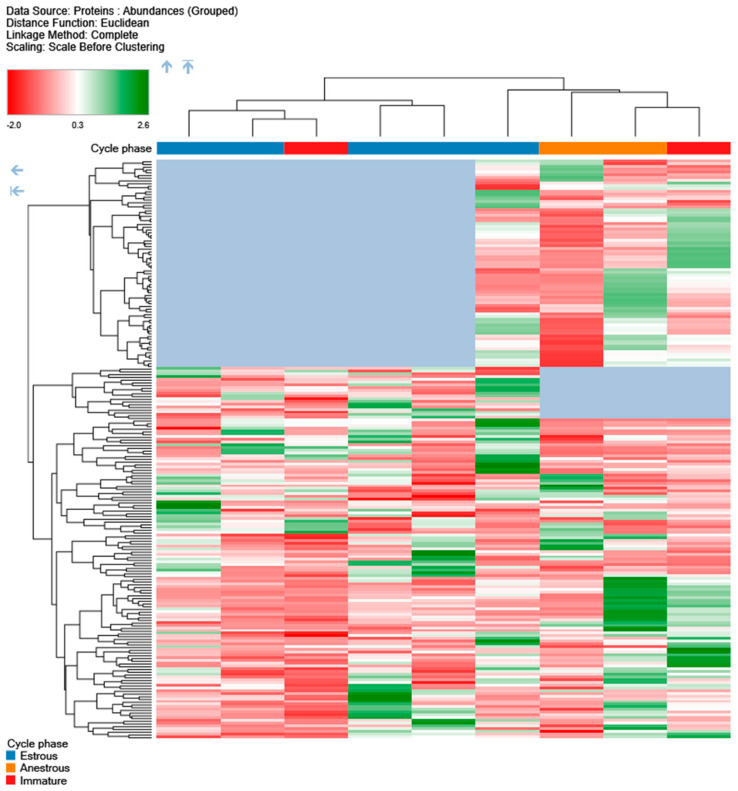
Hierarchical clustering of urine samples. Samples collected during estrus phase are indicated in orange color, and samples from anestrus phase are indicated in blue color, while samples from immature animals are in red.

PCA analysis and hierarchical clustering were performed using standard implementations of these methods, as available in Proteome Discoverer 2.5 software, using all measured samples, excluding pooled control. The inter-relationship of principal components #1 and #2, depicted in Figure 1, showed a clear separation between estrus and anestrus samples along the first principal component, with high dispersion for anestrus samples along the second component. The relationships within protein expression profiles were assessed using the l2 (Euclidean) norm as a distance metric with complete-linkage clustering, with results illustrated in Figure 2.

## 3. Results

A total of 240 proteins were identified in the urine samples. The lists of identified and quantified proteins are provided in Supplementary Table S1a. We observe that our results are robust to the tightening of thresholds, as illustrated in Supplementary Table S1b, which comprises nearly the same proteins as these used in this work, but are identified with 0.05 Da fragment and 10 ppm maximum precursor tolerances. Increasing the threshold of fragment tolerance further to 0.02 Da results in the identification of no meaningful protein signal (as demonstrated in Supplementary Table S1c).

Use of classic evaluation techniques for elucidating signal in the identified proteins has yielded interesting results congruent with the expectations, yet not fully informative (shown in Table 2, Table 3, Table 4 and Table 5). Therefore, PCA analysis (Figure 1) and hierarchical clustering (Figure 2) were performed for the differentiation of specific biological states. Results are presented in the figures below.

From the above figure, it is evident that there is a clear separation between the estrus and anestrus samples. PC1 clearly separates the estrus samples from anestrus ones, and in conjunction with PC2, the separation is even clearer. We also observe that two immature pools (in green) tend to follow the anestrus samples in the PC2 dimension, but reside near opposite limits of the PC1 range in the vicinity of either of the estrus or anestrus groups. This suggests that the proteomic profile of the estrus urine may be convergent; meanwhile, there is a significant dispersion in the case of anestrus samples.

There is a clear separation at the first layer of the tree between the bulk of the estrus samples and the anestrus ones. Both clusterings appear to be influenced by the absence of certain proteins in the estrus and anestrus groups, respectively (indicated in blue).

We performed a BLAST search of known murine MUPs against dog proteomes with an e-value cut-off of 1e-10 to permit the identification of more distant homologs. Naturally, the most relevant hits included lipocalins predominantly present in saliva. However, canine beta-lactoglobulin-1 and beta-lactoglobulin-2 (P33685 and P33686), belonging to the lipocalin family and sharing 90% sequence identity, were identified with high confidence in the mass spectrometry experiment. Both beta-lactoglobulins were identified only in the estrus samples.

Another highly homologous hit to murine MUPs in our results was A0A8P0NKK8 (lipocln_cytosolic_FA-bd_dom domain-containing protein), whose close homologs can be identified uniquely in mammals (predominantly Eutheria) with more distant homologs present in Archelosauria. This protein was found across all samples and pools; its abundance in the estrus pool was 44.3% higher than in the anestrus one (anestrus: estrus ratio: 0.693).

## 4. Discussion

The results of our study show the presence of several proteins in the urine samples which are characteristic of the estrus female. Considering that urine is expected to be relatively devoid of protein with proteinuria being associated with pathology in dogs, the amount of identified proteins in the estrus urine was unexpectedly high.

Changes in the composition of urinary proteins in the context of the estrus/ovarian cycle were investigated in other species. Muthukumar et al. [47] studied urinary proteins in female house rats and evidenced a correlation of lipocalin concentration with the phases of the estrus cycle. In dogs, lipocalin proteins were primarily examined in the context of allergenic agents, and the source used was saliva [48]. The lipocalins are predominantly involved in the transport of small molecules, such as steroids; therefore, it is plausible that they are key molecules in chemical signaling. In fact, the major respiratory allergens of dogs, mice, rats, horses, and cows belong to the this group of proteins [48]. According to our best knowledge, there were no previous studies on identifying estrus-specific proteins in domestic dogs.

Promisingly, we identified proteins belonging to the lipocalin family of canines (beta-lactoglobulin-1 and beta-lactoglobulin-2, (P33685 and P33686, respectively)) in the estrus urine samples. However, due to a high degree of sequence similarity between the two proteins, it was impossible to ascertain which homolog was present in the urine. It is worth noticing that beta-lactoglobulins are small, very stable proteins that contain a hydrophobic pocket, and we postulated them as carriers of semiochemicals in dog urine. It is also interesting that beta-lactoglobulins were absent in the anestrus samples.

It is worth mentioning that A0A8P0NKK8 was present across all the samples and pools. In particular, its abundance (43% higher) in the estrus pool was higher than in the anestrus pool (anestrus: estrus ratio—0.693). Close homologs of this protein are also found among human lipocalins. LCNL1 and LCNL15 (UniProt accession IDs: Q6ZST4 and Q6UWW0) are preferentially expressed in the testes and seminal ducts implicating them in reproductive processes. It could also be the same function the proteins exhibit in canines.

Although they were not yet proven to be related to semiochemical or chemical signals, the other proteins identified in the female urine during estrus (Clusterin, Proenkephalin (PENK), and Liver-expressed antimicrobial peptide 2 (LEAP2)) are also worth focusing on. Clusterin, a heterodimeric glycoprotein, was isolated from the ram’s rete testis fluid that elicited the clustering of Sertoli cells [49]. Clusterin is also produced by various tissues and identified in biological fluids [50]. In dogs, Clusterin was identified concerning kidney and urinary tract disorders, infections, and injuries [51]. Clusterin, on the other hand, is involved in tissue remodeling, immune defense, and the transport of biologically active peptides. Interestingly, Clusterin is also detected in the ovary where it may facilitate sperm–ovum interactions. It is crucial to note the involvement of Clusterin in sperm maturation [50].

Taking into account the presence of Clusterin in many body fluids and a wide array of species, its involvement in the process of semiochemical communication remains elusive. However, Clusterin was found in greater concentrations in follicular fluids than in plasma. It was also suggested that it may likely play a role in follicle physiology and ovarian activity at the pre-ovulatory stage [52]. Clusterin was also identified in the estrus saliva of buffaloes [47]. A study by Zwain et al. [53] proved the association of Clusterin with programmed cell death (apoptosis) and follicular atresia in rats.

In the ovaries and uteri of rodents, the expression of the gene Proenkephalin (PENK) is significantly altered during the estrus cycle, wherein the highest concentrations are found during estrus [54]. Given the changes in the concentration, the association of Proenkephalin within the female reproductive system is expected. However, the interaction between the reproductive hormones (estrogens and progesterone) and Proenkephalin gene expression varies among species [55]. Proenkephalin is cleaved into opioid peptides (met-enkephalin and leu-enkephalin), whose role in nociception is widely studied. The role of the estrus cycle in opioid antinociception in dogs has not been systematically studied. However, similar investigations in rats demonstrate that opioids are least potent during estrus compared to metestrus and proestrus. Konturek et al. [56] showed that enkephalins inhibit pancreatic bicarbonate and protein (somatostatin) secretion during endogenous or exogenous stimulation (secretin or cholecystokinin-octapeptide) in dogs. Therefore, the presence of PENK in urine is expected due to the influence of sex steroid hormones on insulin homeostasis.

Liver-expressed antimicrobial peptide 2 (LEAP2) is a 40-residue cationic peptide that exhibits antimicrobial activity. It is highly expressed in the liver but also produced by other tissues and organs, such as the kidney [57]. It was proposed that antimicrobial peptides (AMPs) in domestic animals have beneficial effects on immune regulation and the reproductive system [58]. LEAP2 is also postulated to be involved in the control of sperm maturation [58]. Furthermore, LEAP2 is an endogenous antagonist of the ghrelin receptor; meanwhile, the spikes of ghrelin expression in estrus are an established phenomenon.

The role of bacteria in the process of synthesis of semiochemical signals has been discussed in many animals. Microbes are potential regulators of chemical signals, as evidenced by their presence in the vaginas of canines [59,60]. It could also be possible that the microbes facilitate the attraction of males toward females through the compounds they synthesize [61,62,63]. In this purview, the presence of infection-associated proteins (GUSB, GZMB, LEAP2, LY6D) in estrus urine is interesting.

Non-volatile compounds, such as proteins, can play various roles in chemical communication. The proteins may themselves act as pheromones or carry specific volatile ligands that act as pheromones. The proteins, in addition, can also be involved in other physiological processes. In cats, a major allergen (Fel d 1-tetrameric glycoprotein of the secretoglobin superfamily) is responsible for binding lipids, similar to the mouse androgen-binding protein [64]. Despite the well-studied antimicrobial function, these peptides can also be crucial in inter-individual communication.

Odorant binding proteins (OBPs) belonging to the lipocalin superfamily can be found both in the main and additional olfactory systems [65], but they also were identified in the glands responsible for the secretion of chemical signals, such as the canine anal sac glands [66]. OBPs are closely homologous to the pheromone carrier proteins (such as allergen Can f 4). The primary function of the mentioned proteins is to bind the pheromone compounds and release them to the environment for manifesting the effect [67,68,69].

Some proteins of this kind were described in the nasal mucus of buffaloes and pigs, suggesting that OBP may bind the odorants for further processing [68]. Despite the apparent function in the process of odor/pheromone binding and transportation, other interesting functions of the OBP have been described. In cattle, Mitchel et al. [70] proved that OBP present in the lung and other parts of respiratory tracts might inhibit neutrophil recruitment by inflammatory mediators, having the ability to bind macrophage-derived inflammatory mediators within the airways. The study performed by Cerna et al. [71] in mice showed the co-expression of OBP with antimicrobial proteins. This confirmed that the same proteins play various roles in the organism. Thus, their presence during some specific physiological conditions could relate to particular events (can be directly involved in reproductive processes, luring, or others) but also can play another role. (Overexpression of the proteins increasing antibacterial defense mechanism is to be expected during the estrus period, which is associated with an increased abundance of microorganisms.) Thus, the presence of proteins seemingly not involved in the pheromone binding does not necessarily imply the lack of importance for the whole process. Moreover, the lack of direct function connecting their role with reproductive processes should not exclude them unequivocally in the pool of characteristics for some period compounds.

The functions of some proteins detected in our study, which are connected with sperm maturation, facilitating sperm–ovum interactions, and antibacterial activity, certainly justify their presence in the urine of females in estrus. However, they do not exclude these proteins from their implication with the compounds potentially involved in communication and attractiveness modulation. Interestingly, a phenomenon of reduced attractiveness after vaginal microflora reduction, which was observed in several species, could suggest the importance of the microflora for creating adequate semiochemical signals [61,62,63]. In dogs, sheep, and rats, reduced vaginal microflora (after antibiotic administration) decreased the sexual attractiveness of treated females [61,62,63]. However, a detailed explanation of this mechanism has not been presented. It is worth evaluating if reducing the vaginal microflora by the use of antibiotics also leads to reducing the antimicrobial peptides in female secretions. If it does, it may be that this phenomenon is involved in modifying the attractiveness of females to the males. In this context, it is worth mentioning acute phase proteins (APPs) in canine urine whose concentration remains unchanged during the reproductive cycle [72].

Despite the proteins belonging to the lipocalin family, the other proteins are also putatively involved in signaling by responding to external stimuli. These facts corroborate the hypothesis that observed proteins might be involved in chemical communication. However, conclusive confirmation of this claim requires further investigation.

## 5. Conclusions

In this paper, we presented the results of the proteomic examination of the urine of female canines during various stages of the ovarian cycle. We found that several proteins were significantly altered during estrus. Interestingly, some of these proteins belong to the lipocalin family; whereas, other proteins identified in the present study are involved in signaling and response to external stimuli. Some of the proteins identified herein are known to be differentially expressed concentrations in the reproductive organs or body fluids (e.g., serum, saliva, etc.) of other species. The association of specific proteins with antimicrobial properties is another promising venue for continued exploration. Furthermore, it is prudent to conduct follow-up studies to evaluate its possible involvement in the chemical communication process. Overall, proteomic evaluation is only a preliminary approach which needs additional evaluation with conspecifics to ascertain the role of proteins in canine chemical communication.

## Figures and Tables

**Table 1:** Samples included in the study.

Individual Samples			Pooled Samples		
Sample Number	Dog	Ovarian Cycle Phase	Sample Number	Dogs	Ovarian Cycle Phase
S1	Female2	Estrus	P1	Female3, Female4	Estrus
S4	Female1	Estrus	P2	Female3, Female4	Anestrus
S5	Female3	Estrus	P3	Female5, Female6	Immature
S6	Female2	Estrus	P4	Female7, Female8	Immature
S7	Female3	Anestrus			

**Table 2:** Results of DAVID analysis. Clusters with an enrichment score of at least 1 were selected with a raw *p*-value and Benjamini–Hofberg adjusted metric (BH *p*-value).

Annotation	Description	Count	*p*-Value	BH *p*-Value
Annotation Cluster 1				
UP_KW_DOMAIN	Signal	8	1.5 × 10^−2^	7.7 × 10^−1^
GOTERM_CC_FAT	extracellular region	6	1.6 × 10^−2^	3.9 × 10^−1^
UP_KW_CELLULAR_COMPONENT	Secreted	4	1.7 × 10^−2^	1.7 × 10^−1^
GOTERM_BP_FAT	response to other organism	3	8.2 × 10^−2^	1.0
GOTERM_BP_FAT	response to external biotic stimulus	3	8.3 × 10^−2^	1.0
GOTERM_BP_FAT	response to biotic stimulus	3	9.1 × 10^−2^	1.0
GOTERM_BP_FAT	response to external stimulus	4	1.2 × 10^−1^	1.0
UP_KW_PTM	Disulfide bond	5	1.4 × 10^−1^	9.7 × 10^−1^
**Annotation Cluster 2**				
GOTERM_CC_FAT	extracellular region	6	1.6 × 10^−2^	3.9 × 10^−1^
GOTERM_BP_FAT	cellular amide metabolic process	4	4.7 × 10^−2^	1.0
GOTERM_CC_FAT	extracellular space	4	7.5 × 10^−1^	1.0
GOTERM_CC_FAT	extracellular region part	4	1.2 × 10^−1^	1.0
UP_KW_PTM	Glycoprotein	3	7.4 × 10^−1^	1.0

**Table 3:** List of proteins with significantly affected abundance rates in estrus vs. anestrus samples (identified as involved in response to external stimuli).

NCBI Gene ID	Protein Name	Relevant GO Term	Abundance Change (Estrus vs. Anestrus)
442971	Clusterin (CLU)	Smoothened signaling pathway GO:0007224	2.153
609750	Liver-expressed antimicrobial peptide 2 (LEAP2)	Antimicrobial humoral immune response mediated by antimicrobial peptide GO:0061844	1000
100687307	Proenkephalin (PENK)	signal transduction GO:0007165 sensory perception GO:0007600	1000

**Table 4:** List of proteins significantly altered in estrus vs. anestrus samples, which were identified as involved in signaling and as secreted/extracellular.

NCBI Gene ID	Protein Name	Relevant GO Term	Abundance Change (Estrus vs. Anestrus)
442971	Clusterin (CLU)	Smoothened signaling pathway GO:0007224	2.153
609750	Liver-expressed antimicrobial peptide 2 (LEAP2)	Antimicrobial humoral immune response mediated by antimicrobial peptide GO:0061844	1000
487447	MBL associated serine protease 2 (MASP2)	cell surface pattern recognition receptor signaling pathway GO:0006958 complement activation, classical pathway GO:0002752 positive regulation of opsonization GO:1903028	1000
100687307	Proenkephalin (PENK)	signal transduction GO:0007165	1000

**Table 5:** List of other proteins significantly altered in estrus vs. anestrus samples.

NCBI Gene ID	Protein Name	Relevant GO Term	Abundance Change (Estrus vs. Anestrus)
403831	Beta glucuronidase (GUSB)	Enzyme. Plays an important role in the degradation of dermatan and keratan sulfates. Present in lysosomes (very much inside the cell, not secreted).	0.432
490630	Granzyme B (GZMB)	Cytosolic enzyme released by lymphocytes to kill virus-infected cells. Not signaling.	2.147
609112	Lymphocyte antigen 6 family member D (LY6D)	Involved in leukocyte differentiation, affected by stilbenoid, present on the cell surface, but not really secreted or signaling. GO:1900740: positive regulation of protein insertion into mitochondrial membrane involved in apoptotic signaling pathway GO:0017148: negative regulation of translation.	1000
489503	Myosin heavy chain 13 (MYH13)	Myosin is a structural protein involved in muscle contractions.	0.396
476279	Sphingomyelin phosphodiesterase acid-like 3A (SMPDL3A)	Enzyme regulating levels of cyclic AMP.	1000

## Data Availability

The data that support the findings of this study are available from the corresponding author (M.D.) upon reasonable request.

## Supplementary Materials

The following supporting information can be downloaded at: https://www.mdpi.com/article/10.3390/vetsci10040292/s1, Table S1a: Identified proteins using the tolerances used in the paper (precursor mass tolerance at 20 ppm, fragment mass tolerance at 0.1 Da); Table S1b: Identified proteins using the more stringent tolerances than in the paper (precursor mass tolerance at 10 ppm, fragment mass tolerance at 0.05 Da). Note that the proteins described in the paper remain deetected with high confidence.; Table S1c: Identified proteins using the stringent tolerances (precursor mass tolerance at 10 ppm, fragment mass tolerance at 0.02 Da). Note lack of detectable proteins with high coverage; Table S2: Composition of TMT 6plexes used in the experiment. Single digit identifiers refer to individual animals, while P# -to pools, in accordance with the labelling in the manuscript. Note that P1 (pool #1) uses the same reference (126) in both plexes.
